# Risk of Adverse Obstetric and Neonatal Outcomes by Maternal Age: Quantifying Individual and Population Level Risk Using Routine UK Maternity Data

**DOI:** 10.1371/journal.pone.0164462

**Published:** 2016-10-07

**Authors:** Laura Oakley, Nicole Penn, Maria Pipi, Eugene Oteng-Ntim, Pat Doyle

**Affiliations:** 1 Department of Non-communicable Disease Epidemiology, London School of Hygiene and Tropical Medicine, London, United Kingdom; 2 King’s College London School of Medicine, London, United Kingdom; 3 Guy’s and St Thomas’ NHS Foundation Trust, London, United Kingdom; UCL Institute of Child Health, University College London, UNITED KINGDOM

## Abstract

**Objective:**

The objective of this study was to investigate whether moderately increased maternal age is associated with obstetric and neonatal outcome in a contemporary population, and to consider the possible role of co-morbidities in explaining any increased risk.

**Study Design:**

Secondary analysis of routinely collected data from a large maternity unit in London, UK. Data were available on 51,225 singleton deliveries (≥22 weeks) occurring to women aged ≥20 between 2004 and 2012. Modified Poisson regression was used to estimate risk ratios for the association between maternal age and obstetric and neonatal outcome (delivery type, postpartum haemorrhage, stillbirth, low birthweight, preterm birth, small for gestational age, neonatal unit admission), using the reference group 20–24 years. Population attributable fractions were calculated to quantify the population impact.

**Results:**

We found an association between increasing maternal age and major postpartum haemorrhage (≥1000ml blood loss) (RR 1.36 95% CI 1.18–1.57 for age 25–29 rising to 2.41 95% CI 2.02–2.88 for age ≥40). Similar trends were observed for caesarean delivery, most notably for elective caesareans (RR 1.64 95% CI 1.36–1.96 for age 25–29 rising to 4.94 95% CI 4.09–5.96 for age ≥40). There was evidence that parity modified this association, with a higher prevalence of elective caesarean delivery in older nulliparous women. Women aged ≥35 were at increased risk of low birthweight and preterm birth. We found no evidence that the risk of stillbirth, small for gestational age, or neonatal unit admission differed by maternal age.

**Conclusions:**

Our results suggest a gradual increase in the risk of caesarean delivery and postpartum haemorrhage from age 25, persisting after taking into account maternal BMI, hypertension and diabetes. The risk of low birthweight and preterm birth was elevated in women over 35. Further research is needed to understand the reasons behind the high prevalence of elective caesarean delivery in nulliparous older mothers.

## Introduction

The average age of mothers at birth in England and Wales has increased steadily from 26.4 years in the mid-1970s to 30.2 in 2014 [1]. One in five births are now to women aged 35 or older, compared to one in ten births two decades ago, with similar trends observed in many other high income countries [2, 3].

Advanced maternal age has long been recognised as a risk factor for poor outcome for both mother and baby [4], associated with an increased risk of perinatal death, pregnancy complications such as diabetes and hypertension, preterm birth, low birth weight, and interventions such as caesarean delivery and induction of labour [5–12]. However, considerable debate remains regarding the point at which maternal age contributes significantly to obstetric or neonatal risk [5, 13, 14] and most previous studies have focused only on ‘advanced’ maternal age (≥35 or ≥40) rather than the effect of moderately increased maternal age. Few studies have investigated the role of conditions and co-morbidities such as obesity, hypertension and diabetes in explaining the increased risk of adverse outcome in older mothers[7, 15, 16].

The aim of our study was to investigate the association between adverse obstetric and neonatal outcomes and increasing maternal age using a recent maternity cohort, and to quantify the population risk associated with increased maternal age.

## Material and Methods

We used routine data collected on all singleton births at ≥22 weeks at Guy’s and St Thomas’ NHS Foundation Trust in London between January 2004 and May 2012. We restricted the sample to women aged ≥20 as very young mothers are at higher risk of certain adverse outcomes [17, 18]. Stillbirths were only included for the analysis focusing on stillbirth as the outcome.

### Variables

Data were extracted from multiple delivery-related variables to create individual binary outcomes; instrumental delivery, emergency caesarean delivery, elective caesarean delivery, major postpartum haemorrhage (PPH) (> = 1000ml estimated blood loss), pre-term delivery (<37 weeks gestation), very preterm delivery (<32 weeks gestation), and stillbirth. Similarly, data were extracted to identify neonates which were low birthweight (<2500g) or admitted to the neonatal unit (NNU) for further care or investigation. A baby was classified as births as small for gestational age (SGA) if they were below the 10^th^ centile for sex and gestation-specific birthweight in this population. For consistency purposes we compared thresholds derived using this method to equivalent cut-offs for term infants in UK growth charts: the results were virtually identical.

The exposure of interest, maternal age, was grouped, and the age group 20–24 used as the reference group as it had the lowest risk profile.

We included a number of other variables as confounders: parity (0, 1, ≥2), mother’s ethnicity (White, Black, South Asian, Other), body mass index (BMI, kg/m^2^) at first contact for antenatal care (underweight (<18.5), recommended (18.5–24), overweight (25–29), and obese (≥30)), smoking status at booking for antenatal care (yes/no), and marital status. Area deprivation was measured using the 2007 index of multiple deprivation (IMD)[19], a classification which uses census data on a variety of economic and social factors. Mothers were categorised according to national IMD quintiles using their postcode at booking.

Binary variables were generated for hypertension (pre-existing or pregnancy related, not including pre-eclampsia) and diabetes (combining pre-existing type 1 and 2 and gestational diabetes).

### Statistical Analysis

We used modified Poisson regression to estimate risk ratios for the association between maternal age category and obstetric and neonatal outcomes, with clustering by women accounted for by the use of robust standard errors. A series of multivariable models were constructed for each outcome. Model 1 adjusted for *a priori* confounders: parity, deprivation, ethnicity, marital status, smoking, and delivery year. Models 2 and 3 additionally adjusted for factors which may act as partial mediators: BMI (model 2), and hypertension and diabetes (model 3). Interaction between maternal age and parity was explored by adding an interaction term to each of the multivariable models. We considered that a p value <0.05 provided evidence of possible interaction. We used a ‘complete case’ sample for all analyses, including only observations with complete data on both the specific outcome under study and all confounders. For variables with >10% missing data, we investigated possible bias and conducted a sensitivity analysis comparing estimates derived from the complete case analysis and those estimated using the full dataset. Analysis was carried out using Stata version 14.

Population attributable fractions were calculated using the formula PAF = p(aRR-1)/aRR, where ‘p’ was the proportion of those with the outcome who were in the relevant age group (i.e. ‘exposed’) and aRR was the adjusted risk ratio. Confidence intervals for PAFs were calculated using the formula suggested by Greenland [20].

### Ethical approval

Ethical approval was granted by the London School of Hygiene and Tropical Medicine Ethics committee. The study was based on routinely collected hospital data. Individual patient consent was not obtained; all patient records were anonymised and de-identified prior to analysis.

## Results

### Description of the study population

Overall, 51,225 births to women aged ≥20 years were included in the study (50,913 livebirths and 312 stillbirths). The mean age of all mothers in the sample was 31.6 years (sd 5.47), and the mean age of primiparous mothers (57% of sample) was 30.8 years (sd 5.37) (Table 1). Over a quarter (28%) of women were 35 years or older, and 6% were aged over 40. One third of women (33%) were Black and 6% were South Asian. White women tended to be slightly older than women from other ethnic backgrounds. BMI values were available for 80% of mothers. Of those with recorded BMI, over one-third of women were overweight or obese (25% overweight, 15% obese), with obesity more prevalent among older women. Overall, there was a high degree of deprivation with 80% of mothers living in the two most deprived quintiles nationally. Older women were more likely to live in the least deprived category: 2% of women aged 20–24 years lived in the two least deprived quintiles and 13% of women aged ≥40). Smoking status at booking was missing for 11% of mothers. Among women with smoking status recorded, few reported smoking (6%). Three-quarters of women were married or cohabiting. The prevalence of hypertension and diabetes was 4.9% and 2.4% respectively; both conditions were more common in older women.

**Table 1:** Maternal characteristics by maternal age category.

		20-24yrs		25-29yrs		30-34yrs		35-39yrs		≥40yrs		ALL	
		n	(%)	n	(%)	n	(%)	n	(%)	n	(%)	n	(%)
ALL WOMEN		7215	(14.1)	12121	(23.7)	17461	(34.1)	11436	(22.3)	2992	(5.8)	51225	
** **	** **	** **	** **	** **	** **	** **	** **	** **	** **	** **	** **		
**Parity**	Nulliparous	4847	(67.7)	7296	(60.6)	10252	(59.1)	5449	(48.0)	1160	(39.3)	29004	(56.7)
	Para 1	1793	(25.0)	3016	(25.1)	4337	(25.0)	3086	(27.2)	763	(25.8)	12995	(25.4)
	Para 2+	563	(7.9)	1795	(14.9)	2855	(16.5)	2883	(25.4)	1059	(35.8)	9155	(17.9)
	*Missing*	*12*	* *	*14*	* *	*17*	* *	*18*	* *	*10*	* *	71	* *
**Deprivation (IMD)**	Quintile 1 (least deprived)	41	(0.6)	214	(1.8)	699	(4.1)	537	(4.8)	138	(4.7)	1629	(3.2)
	Quintile 2	111	(1.6)	444	(3.7)	1314	(7.6)	929	(8.2)	229	(7.8)	3027	(6.0)
	Quintile 3	408	(5.7)	930	(7.8)	2498	(14.5)	1721	(15.2)	407	(13.8)	5964	(11.7)
	Quintile 4	3290	(46.3)	5528	(46.2)	7809	(45.3)	5130	(45.4)	1340	(45.5)	23097	(45.4)
	Quintile 5 (most deprived)	3302	(46.4)	4917	(41.1)	5011	(29.1)	3044	(27.0)	861	(29.2)	17135	(33.7)
	*Missing*	*63*		*88*		*130*		*75*		*17*		373	
**Ethnicity**	White	2812	(39.7)	4940	(41.7)	9648	(56.3)	6649	(59.5)	1560	(53.6)	25609	(50.8)
	Asian	428	(6.0)	916	(7.7)	978	(5.7)	475	(4.3)	114	(3.9)	2911	(5.8)
	Black	2954	(41.7)	4595	(38.8)	4831	(28.2)	3077	(27.6)	1005	(34.6)	16462	(32.6)
	Other	923	(13.0)	1465	(12.4)	1768	(10.3)	1038	(9.3)	256	(8.8)	5450	(10.8)
	*Missing*	*98*	* *	*205*	* *	*236*	* *	*197*	* *	*57*	* *	793	* *
**BMI**	Underweight	306	(5.3)	356	(3.7)	400	(2.9)	203	(2.3)	34	(1.5)	1299	(3.2)
	Recommended weight	3347	(57.6)	5544	(57.1)	8445	(60.5)	5007	(56.4)	1104	(47.9)	23447	(57.2)
	Overweight	1427	(24.6)	2412	(24.8)	3309	(23.7)	2288	(25.8)	670	(29.1)	10106	(24.7)
	Obese	770	(13.3)	1482	(15.3)	1908	(13.7)	1454	(16.4)	522	(22.7)	6136	(15.0)
	*Missing*	*1365*		*2327*		*3399*		*2484*		*662*		10237	
**Marital status**	Married or living with partner	3749	(52.3)	8542	(71.1)	14247	(82.2)	9311	(82.3)	2315	(78.4)	38164	(74.7)
	Single	3457	(48.2)	3553	(29.6)	3174	(18.3)	2074	(18.3)	665	(22.5)	12923	(25.3)
	*Missing*	*9*	* *	*26*	* *	*40*	* *	*51*	* *	*12*	* *	138	
**Smoking**	Non-smoker at booking	5684	(86.9)	10121	(93.4)	14863	(96.3)	9532	(96.2)	2493	(95.9)	42693	(93.6)
	Smoker at booking	893	(13.7)	794	(7.3)	676	(4.4)	445	(4.5)	134	(5.2)	2942	(6.4)
	*Missing*	*638*	* *	*1206*	* *	*1922*	* *	*1459*	* *	*365*	* *	5590	* *
**Hypertensive Disorders**	No hypertension	6904	(96.2)	11560	(96.0)	16681	(96.1)	10835	(95.3)	2751	(92.8)	48731	(95.1)
	Hypertension	311	(4.3)	561	(4.7)	780	(4.5)	601	(5.3)	241	(8.1)	2494	(4.9)
**Diabetes**	No diabetes	7130	(99.4)	11903	(98.8)	17049	(98.2)	11077	(97.5)	2848	(96.1)	50007	(97.6)
	All diabetes	85	(1.2)	218	(1.8)	412	(2.4)	359	(3.2)	144	(4.9)	1218	(2.4)
**Delivery year**	2004–05	1695	(23.6)	2684	(22.3)	3548	(20.4)	2294	(20.2)	572	(19.3)	10793	(21.1)
	2006–07	1781	(24.8)	2856	(23.7)	3886	(22.4)	2598	(22.9)	667	(22.5)	11788	(23.0)
	2008–09	1783	(24.8)	3018	(25.1)	4368	(25.2)	2867	(25.2)	694	(23.4)	12730	(24.9)
	2010–12	1956	(27.3)	3563	(29.6)	5659	(32.6)	3677	(32.4)	1059	(35.7)	15914	(31.1)

### Obstetric outcomes

The prevalence of obstetric outcomes by maternal age is presented in Table 2 and the results of multivariable analysis presented in Table 3. The complete case sample for multivariable analysis was n = 39,625 for outcomes other than stillbirth, and n = 39,863 for the stillbirth analysis. All reported estimates are from model 3 unless otherwise specified. After adjusting for other factors, maternal age was strongly associated with delivery type (Table 3). Increasing maternal age group was associated with a mildly elevated risk of instrumental delivery (RRs between 1.14–1.29), except for a non-significant association in women ≥40 years (Table 3). There was a strong association between maternal age and both emergency and elective caesarean delivery. The risk of emergency caesarean delivery increased linearly with maternal age group (highest RR ≥40 years RR 1.84, 95% CI 1.67–2.03). After adjusting for other factors and compared to women aged 20–24, women aged ≥40 had nearly five times the risk of elective caesarean delivery (RR 4.94, 95% CI 4.09–5.96), and for women aged 35–39 years there was a threefold increase in risk (RR 3.51, 95% CI 2.94–4.18).

**Table 2:** Obstetric and neonatal outcomes by maternal age category.

		20-24yrs		25-29yrs		30-34yrs		35-39yrs		≥40yrs		ALL	
		n	(%)	n	(%)	n	(%)	n	(%)	n	(%)	n	%
**OBSTETRIC OUTCOMES**		** **	** **	** **	** **	** **	** **	** **	** **	** **	** **	** **	** **
**Delivery type**^**1**^	Spontaneous vaginal delivery	4958	(69.6)	7468	(62.5)	9398	(54.5)	5639	(50.0)	1338	(45.4)	28801	(57.0)
	Instrumental delivery	819	(11.5)	1576	(13.2)	2760	(16.0)	1537	(13.6)	289	(9.8)	6981	(13.8)
	Emergency caesarean	1151	(16.2)	2295	(19.2)	3568	(20.7)	2468	(21.9)	702	(23.8)	10184	(20.1)
	Elective caesarean	198	(2.8)	616	(5.2)	1523	(8.8)	1632	(14.5)	617	(20.9)	4586	(9.1)
	*Missing*	*50*		*89*		*115*		*88*		*19*		*361*	
**Stillbirth**	No	7176	(99.5)	12044	(99.4)	17364	(99.4)	11364	(99.4)	2965	(99.1)	50913	(99.4)
	Yes	39	(0.5)	77	(0.6)	97	(0.6)	72	(0.6)	27	(0.9)	312	(0.6)
**Postpartum haemorrhage**^**1**^	None (<500ml)	5169	(75.7)	7981	(70.3)	10814	(66.4)	6834	(64.0)	1624	(59.6)	32422	(67.7)
	Minor (500-999ml)	1345	(19.7)	2670	(23.5)	4234	(26.0)	2904	(27.2)	817	(30.0)	11970	(25.0)
	Moderate (1000-1999ml)	280	(4.1)	587	(5.2)	1034	(6.4)	770	(7.2)	217	(8.0)	2888	(6.0)
	Severe (≥2000ml+)	36	(0.5)	11	(0.1)	201	(1.2)	168	(1.6)	65	(2.4)	481	(1.0)
	*Missing*	*346*		*685*		*1081*		*688*		*242*		*3042*	
**Gestation**^**1**^	<32 weeks	88	(1.3)	168	(1.5)	209	(1.3)	155	(1.4)	44	(1.6)	664	(1.4)
	32–36 weeks	362	(5.3)	546	(4.8)	722	(4.4)	528	(4.9)	193	(7.0)	2351	(4.9)
	≥37–41 weeks	6080	(88.5)	10123	(88.8)	14535	(88.8)	9512	(88.6)	2388	(87.0)	42638	(88.6)
	≥42 weeks	339	(4.9)	569	(5.0)	906	(5.5)	536	(5.0)	121	(4.4)	2471
	*Missing*	*307*	* *	*638*	* *	*992*	* *	*633*	* *	*219*	* *	*2789*	
**NEONATAL OUTCOMES**													
**Small for gestational age**^**1**^	Yes	890	(13.0)	1217	(10.7)	1351	(8.3)	841	(7.8)	239	(8.7)	4538	(9.4)
	No	5968	(86.9)	10183	(89.3)	15016	(91.8)	9889	(92.2)	2506	(91.3)	43562	(90.6)
	*Missing*	*318*	* *	*644*	* *	*997*	* *	*634*	* *	*220*	* *	*2813*	
**Low birthweight**^**1**^	Yes	506	(7.4)	735	(6.4)	853	(5.2)	662	(6.2)	206	(7.5)	2962	(6.2)
	No	6360	(92.6)	10669	(93.6)	15511	(94.8)	10066	(93.8)	2538	(92.5)	45144	(93.8)
	*Missing*	*310*		*640*		*1000*		*636*		*221*		*2807*	
**NNU admission**^**1**^	Yes	433	(6.0)	672	(5.6)	835	(4.8)	659	(5.8)	192	(6.5)	2791	(5.5)
	No	6734	(94.0)	11361	(94.4)	16509	(95.2)	10693	(94.2)	2769	(93.5)	48066	(94.5)
	*Missing*	*9*	* *	*11*	* *	*20*	* *	*12*	* *	*4*	* *	*56*	* *

^1^Only reported for livebirths (n = 50,913).

**Table 3:** Unadjusted and adjusted risk ratios for the association between maternal age and obstetric and neonatal outcome^1^.

	25-29yrs				30-34yrs				35-39yrs				≥40yrs			
	Unadj.	Model 1^a^	Model 2^b^	Model 3^c^	Unadj.	Model 1^a^	Model 2^b^	Model 3^c^	Unadj.	Model 1^a^	Model 2^b^	Model 3^c^	Unadj.	Model 1^a^	Model 2^b^	Model 3^c^
	(95% CI)	(95% CI)	(95% CI)	(95% CI)	(95% CI)	(95% CI)	(95% CI)	(95% CI)	(95% CI)	(95% CI)	(95% CI)	(95% CI)	(95% CI)	(95% CI)	(95% CI)	(95% CI)
**OBSTETRIC OUTCOMES**	** **	** **				** **			** **	** **				** **		
Instrumental delivery	1.11*	1.13**	1.14**	1.14**	1.36***	1.28***	1.29***	1.29***	1.18***	1.27***	1.29***	1.28***	0.85*	1.09	1.11	1.11
	(1.02–1.22)	(1.04–1.24)	(1.04–1.25)	(1.04–1.25)	(1.25–1.48)	(1.18–1.39)	(1.19–1.40)	(1.18–1.40)	(1.08–1.29)	(1.16–1.39)	(1.17–1.41)	(1.17–1.41)	(0.74–0.98)	(0.94–1.25)	(0.96–1.28)	(0.96–1.28)
Emergency caesarean	1.19***	1.30***	1.28***	1.27***	1.28***	1.54***	1.50***	1.49***	1.37***	1.82***	1.75***	1.71***	1.44***	2.03***	1.91***	1.84***
	(1.11–1.29)	(1.21–1.40)	(1.19–1.38)	(1.18–1.37)	(1.20–1.38)	(1.44–1.66)	(1.40–1.62)	(1.39–1.60)	(1.27–1.47)	(1.69–1.96)	(1.62–1.89)	(1.59–1.85)	(1.31–1.59)	(1.84–2.25)	(1.73–2.11)	(1.67–2.03)
Elective caesarean	1.81***	1.66***	1.64***	1.64***	2.84***	2.49***	2.46***	2.43***	4.52***	3.66***	3.59***	3.51***	7.05***	5.34***	5.13***	4.94***
	(1.51–2.16)	(1.39–1.99)	(1.37–1.97)	(1.36–1.96)	(2.40–3.36)	(2.09–2.96)	(2.07–2.92)	(2.04–2.88)	(3.82–5.34)	(3.08–4.36)	(3.01–4.27)	(2.94–4.18)	(5.90–8.44)	(4.43–6.44)	(4.25–6.18)	(4.09–5.96)
Stillbirth	1.24	1.22	1.20	1.19	1.20	1.29	1.26	1.25	1.34	1.40	1.36	1.32	2.05*	1.95*	1.86*	1.75
	(0.78–1.97)	(0.76–1.93)	(0.76–1.91)	(0.75–1.90)	(0.77–1.87)	(0.82–2.05)	(0.80–2.00)	(0.79–1.97)	(0.85–2.12)	(0.86–2.28)	(0.84–2.21)	(0.81–2.14)	(1.17–3.60)	(1.07–3.58)	(1.01–3.41)	(0.95–3.20)
Major PPH (≥1000ml)	1.37***	1.37***	1.36***	1.36***	1.72***	1.77***	1.74***	1.73***	2.01***	2.16***	2.10***	2.08***	2.41***	2.57***	2.45***	2.41***
	(1.18–1.58)	(1.19–1.59)	(1.18–1.57)	(1.18–1.57)	(1.50–1.96)	(1.54–2.03)	(1.52–2.00)	(1.51–1.99)	(1.75–2.31)	(1.87–2.49)	(1.82–2.42)	(1.80–2.40)	(2.03–2.86)	(2.16–3.06)	(2.05–2.92)	(2.02–2.88)
Preterm birth (<37w)	0.96	1.06	1.06	1.04	0.87*	1.10	1.09	1.06	1.05	1.35***	1.33***	1.27**	1.37***	1.69***	1.64***	1.50***
	(0.83–1.09)	(0.93–1.22)	(0.92–1.21)	(0.91–1.19)	(0.77–0.99)	(0.96–1.26)	(0.95–1.24)	(0.93–1.22)	(0.92–1.21)	(1.17–1.56)	(1.15–1.53)	(1.10–1.46)	(1.14–1.64)	(1.40–2.04)	(1.36–1.98)	(1.24–1.81)
Very preterm birth (<32w)	1.06	1.24	1.24	1.22	0.91	1.34	1.33	1.31	1.11	1.70**	1.68**	1.59**	1.17	1.68*	1.64*	1.48
	(0.77–1.45)	(0.91–1.70)	(0.90–1.69)	(0.89–1.67)	(0.67–1.24)	(0.98–1.84)	(0.97–1.82)	(0.95–1.79)	(0.81–1.52)	(1.22–2.37)	(1.20–2.34)	(1.14–2.23)	(0.75–1.83)	(1.05–2.68)	(1.03–2.63)	(0.92–2.38)
**NEONATAL OUTCOMES**	** **	** **	** **		** **	** **	** **		** **	** **	** **		** **	** **	** **	
SGA	0.81***	0.90*	0.92	0.92	0.64***	0.82***	0.84***	0.84***	0.60***	0.82***	0.85**	0.85**	0.69***	0.94	1.00	0.98
	(0.74–0.89)	(0.82–0.99)	(0.84–1.01)	(0.84–1.01)	(0.58–0.70)	(0.74–0.90)	(0.76–0.93)	(0.76–0.93)	(0.54–0.67)	(0.74–0.92)	(0.77–0.95)	(0.76–0.95)	(0.59–0.80)	(0.81–1.11)	(0.85–1.17)	(0.84–1.15)
Low birthweight	0.87*	1.01	1.03	1.02	0.71***	0.97	0.99	0.98	0.84*	1.22***	1.25**	1.20*	1.09	1.52***	1.57***	1.43***
	(0.89–1.15)	(0.89–1.15)	(0.90–1.17)	(0.89–1.16)	(0.62–0.80)	(0.85–1.11)	(0.87–1.14)	(0.86–1.12)	(0.74–0.96)	(1.06–1.40)	(1.09–1.44)	(1.04–1.38)	(0.91–1.31)	(1.26–1.83)	(1.30–1.89)	(1.19–1.73)
NNU admission	0.88	0.88	0.87*	0.86*	0.77***	0.75***	0.74***	0.73***	0.96	0.94	0.91	0.89	0.99	0.97	0.93	0.87
	(0.77–1.00)	(0.77–1.00)	(0.76–0.99)	(0.75–0.99)	(0.67–0.87)	(0.66–0.87)	(0.76–0.99)	(0.64–0.84)	(0.84–1.10)	(0.81–1.09)	(0.79–1.06)	(0.77–1.03)	(0.82–1.20)	(0.79–1.19)	(0.76–1.14)	(0.71–1.07)

^1^Eligible complete case sample for all outcomes other than stillbirth n = 39,625 (minus observations missing information on the specified outcome); complete case sample for stillbirth n = 39,863.

^a^Adjusted for parity, deprivation, ethnicity, marital status, smoking and year of delivery

^b^Adjusted for parity, deprivation, ethnicity, marital status, smoking, year of delivery and BMI

^c^Adjusted for parity, deprivation, ethnicity, marital status, smoking, year of delivery, BMI, hypertension and diabetes

*p <0.05

**p <0.005

***p<0.001.

Major PPH was independently associated with older maternal age group with a dose-response trend (test for trend p <0.001). For women aged 40 and over, the risk ratio for major PPH was 2.41 (95% CI 2.02–2.88). Although stillbirth was more prevalent in older age groups, this trend was not significant. Both preterm and very preterm birth showed a slightly elevated risk in women aged 35 and over compared to women 20–24, although the increased risk was not significant for very preterm birth in women ≥40.

### Neonatal outcomes

Association between maternal age and neonatal outcomes was less clear. Small for gestational age (SGA) showed an inconsistent association with maternal age, with women aged 30–39 years having a lower adjusted risk of SGA compared to women aged 20–24 years (Table 3; 30–34 years RR 0.84, 95% CI 0.76–0.93). The risk of low birth weight was significantly higher for women aged 35 and over (35–39 years RR 1.20, 95% CI 1.04–1.38; ≥40 years RR 1.43, 95% CI 1.19–1.73). There was no evidence to suggest that children born to older mothers were more likely to be admitted to a neonatal unit after birth.

### The role of mediating factors

We explored the role of obesity, hypertension and diabetes in our analysis by comparing models with and without adjustment for these factors. Overall, there was little difference between estimates derived from the main models (model 3) which included these variables, and estimates from models excluding these variables (models 1 and 2).

### Interaction between maternal age and parity

**Fig 1:**
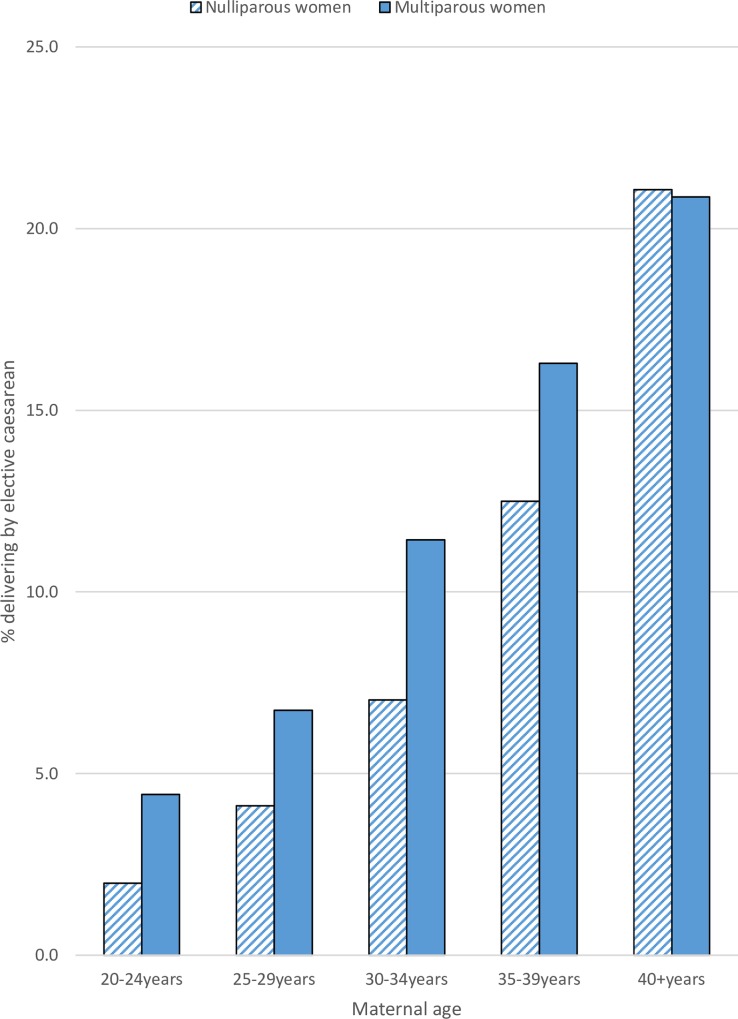
Elective caesarean deliveries by parity and maternal age.

There was evidence to suggest that the association between maternal age and elective caesarean delivery was modified by parity (Fig 1). At younger maternal age, elective caesarean deliveries were more prevalent in multiparous women. As maternal age increased, the risk of elective caesarean delivery rose more sharply in nulliparous women (Table 4) (35-39yrs nulliparous RR 4.67 95% 3.61–6.03, multiparous RR 2.85 95% CI 2.27–3.58; ≥40yrs nulliparous RR 8.23 95% CI 6.23–10.88, multiparous RR 3.73 95% CI 2.94–4.74; test for interaction p value <0.001). There was no evidence of interaction between parity and maternal age for other outcomes.

**Table 4:** Interaction between maternal age and parity.

		25-29yrs	30-34yrs	35-39yrs	≥40yrs
		Adjusted RR^1^	Adjusted RR^1^	Adjusted RR^1^	Adjusted RR^1^
		(95% CI)	(95% CI)	(95% CI)	(95% CI)
**Elective caesarean**					
	Primiparous	1.94***	2.69***	4.67***	8.23***
		(1.48–2.54)	(2.08–3.47)	(3.61–6.03)	(6.23–10.88)
	Multiparous	1.40*	2.19***	2.85***	3.73***
		(1.10–1.78)	(1.74–2.75)	(2.27–3.58)	(2.94–4.74)

^1^Adjusted for parity, deprivation, ethnicity, BMI, marital status, smoking, year of delivery, hypertension and diabetes (Model 3)

* p <0.05

**p <0.005

***p<0.001.

### Population attributable fractions (PAF)

PAFs take into account both the strength of association between maternal age and adverse outcome (the risk ratios) and the distribution of births by maternal age in the population. Estimated PAFs are presented in Table 5 and can be interpreted as the degree of ‘excess’ obstetric risk in this population that may be preventable if women in these age groups faced the same level of risk as those aged 20–24 having adjusted for parity, BMI, hypertension, diabetes and other confounding factors.

**Table 5:** Population attributable fractions for obstetric and neonatal outcomes by maternal age.

	25-29yrs	30-34yrs	35-39yrs	≥40yrs
	PAF	PAF	PAF	PAF
	(95% CI)	(95% CI)	(95% CI)	(95% CI)
**OBSTETRIC OUTCOMES**	** **	** **	** **	** **
Instrumental delivery	2.8%	8.9%	4.7%	0.4%
	(1.0, 4.5)	(6.1, 11.6)	(3.0, 6.5)	(-0.2, 1.0)
Emergency caesarean	4.9%	11.6%	9.9%	3.0%
	(3.4, 6.3)	(9.7, 13.5)	(8.5, 11.2)	(2.4, 3.6)
Elective caesarean	5.7%	19.6%	24.0%	10.9%
	(3.8, 7.7)	(16.6, 22.4)	(21.5, 26.3)	(9.5, 12.2)
Stillbirth	3.8%	6.6%	5.6%	4.0%
	(-6.5, 13.0)	(-7.1, 18.5)	(-4.2, 14.5)	(-0.6, 8.3)
Major PPH (≥1000ml)	5.3%	15.2%	13.9%	4.8%
	(2.9, 7.7)	(11.8, 18.5)	(11.5, 16.4)	(3.6, 5.9)
Preterm birth (<37w)	0.9%	1.7%	5.0%	2.6%
	(-2.3, 4.0)	(-2.3, 5.6)	(2.0, 7.9)	(1.3, 3.9)
Very preterm birth (<32w)	4.5%	7.3%	8.8%	2.1%
	(-2.7, 11.2)	(-1.2, 15.0)	(2.5, 14.7)	(-0.7, 4.7)
**NEONATAL OUTCOMES**				
SGA	-2.3%	-5.7%	-3.2%	-0.1%
	(-5.0, 0.3)	(-9.1, -2.5)	(-5.4, -1.0)	(-0.9, 0.7)
Low birthweight	0.5%	-0.6%	3.6%	2.2%
	(-2.7, 3.6)	(-4.5, 3.1)	(0.9, 6.4)	(0.9, 3.4)
NNU admission	-3.9%	-11.0%	-2.9%	-1.0%
	(-7.3, -0.6)	(-16.1, -6.2)	(-6.6, 0.6)	(-2.2, 0.3)

The PAF for elective caesarean delivery among women aged ≥40 was 10.9%, and 24% for women aged 35–39. Crudely summing these PAFS suggests that six in ten elective caesareans could be attributed to maternal age above the baseline of 20–24 years. For major PPH it was the estimated PAF for women aged 35–39 was 13.9% and 4.8% for women ≥40, and the overall burden of major PPH attributable to maternal age above the baseline of 20–24 years was estimated to be in the region of ~40%. While there is a degree of variability in the calculated PAFS, confidence intervals for emergency caesarean, elective caesarean and major PPH did not cross one.

### Sensitivity analysis

We compared estimates of effect in the full sample (i.e. no exclusions for missing BMI and smoking status) and the restricted sample used in our main analysis, adjusting for all variables with <2% missing data. The results of this sensitivity analysis are presented as supplementary data (S1 Table), and confirm little difference in estimates derived from the full and restricted sample.

## Discussion

### Principal findings

For many of the obstetric outcomes investigated in this study, there was a clear increase in risk beginning from age 25–29. This trend was particularly strong for the risk of elective caesarean delivery (2.8% in women aged 20–24, 20.9% in women ≥40) and major PPH (4.6% in women aged 20–24, 10.4% in women ≥40). The risk of elective caesarean delivery differed by parity: for younger women the risks were similar regardless of parity, but for older women, nulliparous women had a proportionately higher risk of caesarean delivery when compared to multiparous women. There was a significantly elevated risk of preterm birth and low birth weight in women 35 and older. There was no evidence for association between SGA and NNU admission and increasing age.

### Strengths and weaknesses

This analysis is based on a large contemporary cohort of births with information on confounding factors including parity, BMI and area deprivation. We were able to examine the role of hypertension and diabetes in the association between increased maternal age and adverse outcome, which few previous studies have addressed. In addition, we calculated PAFs to demonstrate the public health impact of advancing maternal age on obstetric and neonatal outcomes in this population. To our knowledge these have not been estimated in any previous UK study.

Although our study sample was large, we included only births from one maternity unit in inner London. The location of the unit in an ethnically diverse area with high levels of deprivation may affect the generalisability of our findings. Missing and incomplete information is a common limitation of using routine data. In our study based there was considerable missing data for maternal BMI (20%) and smoking (10%). Women with missing data were shown to be younger. However, the absolute difference was small and sensitivity analysis demonstrated that the risk estimates were not substantially altered when only women for whom BMI and smoking status had been recorded were included. No information was available on whether pregnancy was spontaneously conceived. The use of assisted reproductive techniques (ART) such as IVF rises with age. There is evidence that ART conception is independently associated with a number of adverse outcomes including preterm birth, low birthweight, NNU admission and perinatal mortality, even after adjustment for parity [21]; [22]. [7, 15]Indicators of obstetric risk (such as history of previous deliveries) and information on individual-level socio-economic indicators were also unavailable. Lastly, postpartum haemorrhage was derived from best estimates of blood loss, but blood loss is known to be difficult to estimate accurately [23] and a degree of misclassification is likely.

### Interpretation

The very high rates of caesarean section among older mothers seen here has previously been reported [8–10, 12, 16, 24–26]. Consistent with the findings reported here, a number of previous studies also found that this association differs by parity, with the prevalence of delivery by caesarean highest in nulliparous women [9, 10, 24, 26]}. The strength and consistency of the association between caesarean delivery and increased maternal age has led some authors to suggest that a biological explanation is likely for the increased risk [27]. This may partially explain only the association with emergency caesarean section. Instead, it has been suggested that the surprisingly high risk of elective caesarean delivery among older women is partly attributable to differences in care and maternal preference [28, 29]. It is worth noting that during the time period this study was carried out there was no local policy in place recommending elective caesarean delivery for older women.

A number of previous studies have concluded that parity is an effect modifier of the association between increased maternal age and adverse birth outcomes such as preterm birth and low birthweight [30–32]. We found strong evidence of such interaction only for the analysis focusing on delivery method as an outcome. However, there is some weak evidence from our analyses to suggest that the association between increased maternal age and both low birthweight and preterm birth was stronger in primiparous women.

We found that the risk of PPH increased with maternal age, similar to findings from an earlier study using routine maternity data [8]. As caesarean delivery is known to be a risk factor for PPH [33], it is likely that some of the elevated risk of PPH can be attributed to higher rates of caesarean delivery among older women. Notably, the age specific risks of major PPH were over three times higher than those observed in an earlier study based on births between 1988–1997 in the London North Thames Region [8]. This is consistent with growing evidence suggesting that the risk of PPH has increased over time in a number of high-income countries [34].

Previous studies have observed an increased risk of stillbirth in older mothers [6, 35, 36]. Although stillbirth was more common among older mothers in our study, this trend was not significant. The findings relating to SGA, low birthweight and admission to NNU were inconclusive, suggesting no strong trend in these outcomes by maternal age in the selected population.

Hypertension and diabetes are more common among older mothers and could be considered as potential mediators of the association between older age and adverse outcome. We found very little difference between estimates adjusting for hypertension and diabetes, and those that did not. In the absence of a full mediation analysis, these results suggest that obesity, hypertension and diabetes are likely to have only a minor role in explaining the association between maternal age and adverse outcome in our study population. We conducted a further sensitivity analysis restricting the sample to women without diabetes and hypertension (results not reported). The results from this additional analysis were virtually identical in both trend and magnitude. Overall, our findings support those of a previous UK study using routine maternity data which reported that risk persisted after adjustment for diabetes and pre-eclampsia[8], and those of a UK cohort study which found maternal age was associated with adverse outcome even in a ‘low risk’ population [37].

### Implications

The debate over the appropriate cut-off for obstetric risk and maternal age appears commonly in the literature [6, 38]. Our data describes advancing maternal age as a continuum of increasing risks and reflects similar findings in several other studies [9, 37, 39]. Rather than a simple focus on mothers over a given age, closer attention should be paid to the shifting distribution curve of maternal age. Decisions about timing of childbearing are affected by a plurality of factors and the perceived probable gains in delaying motherhood, such as improved financial security, may outweigh any increase in adverse obstetric risk. Whether maternal age is a modifiable risk factor is the subject of much debate, particularly as there are clearly modifiable risk factors such as obesity which are likely to have a greater impact in public health terms [40].

## Conclusions

Although outcomes among older mothers are generally favourable, we found a consistent association between increasing maternal age and both caesarean delivery (emergency and elective) and PPH beginning from age 25 onwards. There was also evidence of an increased risk of low birth weight and preterm birth in women over 35. The individual and societal costs of these trends are likely to increase with an ageing reproductive population.

## Supporting Information

S1 Table Sensitivity analysis. (DOCX)
