# Supplementary material for: Risk of Adverse Obstetric and Neonatal Outcomes by Maternal Age: Quantifying Individual and Population Level Risk Using Routine UK Maternity Data
Source: PLoS One. 2016 Oct 7;11(10):e0164462. doi: 10.1371/journal.pone.0164462 (PMC5055305; doi:10.1371/journal.pone.0164462)
Supplement: S1 Table — (DOCX) [file pone.0164462.s001.docx]

**SUPPLEMENTARY TABLES**

Table S1. Sensitivity analysis

| ^1^Restricted sample, **excluding** women for whom BMI and smoking status was missing. Eligible complete case sample for all outcomes other than stillbirth n=39,625 (minus observations missing information on the specified outcome); complete case sample for stillbirth n=39,863. |
| --- |
| ^2^Full sample, including women for whom BMI and smoking status was missing. Eligible complete case sample for all outcomes other than stillbirth n=49,567 (minus observations missing information on the specified outcome); complete case sample for stillbirth n=50,913. |
| ^3^Adjusted for parity, deprivation, ethnicity, marital status, year of delivery, hypertension and diabetes |
